# Omicron COVID-19 variant outcomes and vaccination in non-severe and non-critical patients at admission

**DOI:** 10.3389/fpubh.2022.974986

**Published:** 2023-02-09

**Authors:** Hong Zhao, Wenyi Ye, Xia Yu, Yu Shi, Jifang Sheng

**Affiliations:** ^1^State Key Laboratory for the Diagnosis and Treatment of Infectious Diseases, Department of Infectious Diseases, Collaborative Innovation Center for the Diagnosis and Treatment of Infectious Diseases, National Clinical Research Center for Infectious Diseases, The First Affiliated Hospital, School of Medicine, Zhejiang University, Hangzhou, China; ^2^Department of Traditional Chinese Internal Medicine, The First Affiliated Hospital of Zhejiang Chinese Medical University, Hangzhou, China

**Keywords:** COVID-19, Omicron variant, clinical outcome, vaccine dose, non-severe and non-critical patients

## Abstract

The clinical data of patients infected with the Omicron variant virus in Zhejiang Province from January to 14 May 2022 were collected retrospectively. We analyzed the differences in symptoms, clinical categories of COVID-19, length of hospital stay, and time for clearance of Omicron variant viral RNA in the sputum among the groups receiving a different number of vaccine doses. The analysis showed that as the number of vaccine doses increased, the frequency of clinical symptoms, such as fever and fatigue, decreased and the frequency of patients with moderate infections gradually decreased. At the same time, the length of hospital stay was significantly shortened. Based on the multivariate analysis, one vaccine dose [odds ratio (OR): 0.21, 95% confidence interval (CI): 0.08–0.56, *p* = 0.002], two vaccine doses (OR: 0.54, 95% CI: 0.33–0.88, *p* = 0.013), and three vaccine doses (OR: 0.40, 95% CI: 0.24–0.64, *p* < 0.001) shortened the length of hospitalization than those with no vaccination. The persistence of the virus in the sputum was significantly shortened with one vaccine dose (OR: 0.36, 95% CI: 0.15–0.89, *p* = 0.027), two vaccine doses (OR: 0.46, 95% CI: 0.27–0.78, *p* = 0.004), and three vaccine doses (OR: 0.38, 95% CI: 0.22–0.64, *p* < 0.001) than those with no vaccination. Therefore, we concluded that vaccination was an effective way to protect people against infection with the Omicron variant. Indeed, on the premise of the current routine recommendation of vaccination, three vaccines were necessary for people to be protected against the Omicron variant.

## Introduction

Coronavirus 2019 (COVID-19) is a global pandemic (1, 2). Although China successfully contained the transmission of COVID-19 from 2020 to 2021, the emerging Omicron variant is highly transmissible and has outcompeted most containment efforts in Western countries. This finding has raised serious concerns in China in recent months.

According to the Hong Kong University Medicine School of Public Health, the mortality rate was higher in the USA and Singapore (0.38 and 0.17, respectively) (3). As reported by Gupta et al., the Omicron variant causes breakthrough infections (4, 5). Specifically, 91.3% of the population in Hong Kong received one vaccine dose and 81.3% received two vaccine doses. Greater than one-third of the population in Hong Kong has received three vaccine doses (3). An exceedingly high mortality rate occurred in the fifth wave of COVID-19 in Hong Kong; 95.8% of deaths occurred in the elderly who were >60 years of age and ~90% of those who died did not receive two doses of the vaccine (3). In the recent 3-month outbreak epidemic of the Omicron variant in Shanghai, there were >580,000 patients who were infected and >500 deaths (6). Greater than 30 million people completed enhanced immunizations by March, and the vaccination coverage in those >60 and 80 years of age was 83.2 and 48.6%, respectively (6). The efficacy of the vaccine against the Omicron virus infection has not been established, especially among non-severe and non-critical patients with COVID-19. In the current study, we analyzed the clinical data of patients with Omicron variant infection among a retrospective cohort of 5,713 hospitalized patients in the Zhejiang Province of China and determined the potential effect of different doses of vaccine on outcomes.

## Methods

The clinical data of patients with Omicron variant infection in Zhejiang Province from 31 January 2022 to 14 May 2022 were collected, including demographic characteristics and clinical and laboratory examination data during hospitalization in the isolation wards and Fangcang hospitals. Omicron COVID-19 variant-infected individuals were identified using a reverse transcription PCR and the subsequent genomic sequencing. Asymptomatic, mild, moderate, severe, and critically ill patients were defined as previously described (7–9). Subjects who received other types of vaccines were excluded. All hospitalized participants received a standard treatment based on the 9th edition of the Diagnosis and Treatment of COVID-19 by the National Health Commission of China. Two researchers verified the data recorded using Excel software. The study complied with the Helsinki Declaration and was approved by the Ethics Committee of the First Affiliated Hospital of Zhejiang University School of Medicine.

Continuous variables are expressed as the mean ± standard deviation (SD), and qualitative variables are expressed as numbers and percentages. SPSS software (version 26.0; SPSS, Inc., Chicago, IL, USA) was used for the data analysis. The chi-square analysis was used to compare categorical variables, two independent sample *t*-tests were used to compare quantitative variables, and non-parametric tests of k-independent samples were used for multiple-comparison quantitative variables. Univariate and multivariate logistic regression analyses were used to determine the risk factors for host susceptibility to prolonged hospitalization and the persistence of the virus in the sputum. A forest map for the regression analysis was performed using GraphPad Prism for Windows (version 8.0.0; GraphPad Software, San Diego, CA, USA). For the multivariate analysis, the entry and removal probability for the stepwise analysis was set at 0.05 and 0.10, respectively, and variables with a *P*-value of < 0.05 were retained in the final model. The significance level was set at a *p*-value of < 0.05 in all analyses.

## Results

A total of 5,173 COVID-19 patients infected with the Omicron variant were included in the analysis, including 452 (8.7%) who were not vaccinated, 194 (3.8%) who received one dose, 1,767 (34.2%) who received two doses, and 2,760 (53.4%) who received three doses. There were 1,928 (37.3%) patients with asymptomatic infections, 3,044 (58.8%) patients with mild infections, 200 (3.9%) patients with moderate infections, and one (0.0%) patient with a severe infection. As shown in Table 1, the infected individuals with three doses of vaccination were older (*p* < 0.001), hospitalization was shorter (*p* < 0.001), and the clearance of sputum RNA was more rapid (*p* = 0.010).

**Table 1:** Demographic, history, clinical symptoms, and clinical types of different doses of vaccine for COVID-19 infected with Omicron variant.

**Variables**	**Total (%)** **(*N* = 5,173)**	**Doses of vaccine**			
		**0 dose** **(*****n*** = **452)**	**1 dose** **(*****n*** = **194)**	**2 doses** **(*****n*** = **1,767)**	**3 doses** **(*****n*** = **2,760)**
Age (years)^##^	38.80 ± 15.16	37.28 ± 23.09	33.98 ± 14.60^&^	34.90 ± 14.99^$^	41.88 ± 12.80^▴▴^
**Gender (%)**					
Male	3,055 (59.1%)	234 (51.8%)	132 (68.0%)	1,056 (59.8%)	1,633 (59.2%)
Female	2,118 (40.9%)	218 (48.2%)	62 (32.0%)	711 (40.2%)	1,127 (40.8%)
**Comorbidities**					
Hypertension	326 (6.3%)	45 (10.0%)	19 (10.0%)	125 (7.1%)	137(5.0%)
Diabetes	129 (2.5%)	11 (2.4%)	5 (2.6%)	18 (1.0%)	95 (3.4%)
**Symptoms**					
Cough^#^	1,284 (24.8%)	104 (23.0%)	29 (14.9%)	4,333 (24.5%)	718 (26.0%)
Pharyngalgia	883 (17.1%)	63 (13.9%)	32 (16.5%)	292 (16.5%)	496 (18.0%)
Fever^##^	1,280 (24.7%)	131 (29.0%)	45 (23.2%)	494 (28.0%)	610 (22.1%)
Fatigue^#^	435 (8.4%)	52 (11.5%)	21 (10.8%)	159 (9.0%)	203 (7.4%)
Expectoration	503 (9.7%)	32 (7.1%)	13 (6.7%)	189 (10.7%)	269 (9.7%)
Headache	190 (3.7%)	19 (4.2%)	10 (5.2%)	69 (3.9%)	92 (3.3%)
Rhinobyon^#^	482 (9.3%)	27 (6.0%)	14 (7.2%)	146 (8.3%)	295 (10.7%)
Muscle soreness^#^	188 (3.6%)	15 (3.3%)	9 (4.6%)	82 (4.6%)	82 (3.0%)
**Clinical types** ^#^					
Asymptome	1,928 (37.3%)	175 (38.7%)	83 (42.8%)	626 (35.4%)	1,044 (37.8%)
Mild	3,044 (58.8%)	257 (56.9%)	103 (53.1%)	1,053 (59.6%)	1,631 (59.1%)
Ordinary	200 (3.9%)	19 (4.2%)	8 (4.1%)	88 (5.0%)	85 (3.1%)
Severe	1 (0.0%)	1 (0.2%)	0	0	0
Hospitalization rate	3,592 (69.4%)	345 (76.3%)	136 (70.1%)	1,200 (67.9%)^$^	1,711 (62.0%)^▴^
Time during hospitalization (days)^##^	13.71 ± 6.89	16.60 ± 7.83	12.28 ± 5.66^&^	14.38 ± 7.21^$^	12.75 ± 6.32^▴▴^
Time during positive of Omicron variant viral RNA in sputum (days)^#^	10.75 ± 5.64	12.41 ± 6.64	10.49 ± 4.40	11.04 ± 5.76	10.34 ± 5.45^▴^

^#^p < 0.05 or ^*##*^p < 0.001; in differences among different doses of vaccine.

^&^p < 0.05.

^*$*^p < 0.05.

^▴^p < 0.05 or ^▴▴^p < 0.001; differences between zero dose of vaccine and 3 dose of vaccine.

The data are expressed as mean ± standard deviation (SD), or number (percent). Comparisons among different doses of vaccine were performed by the Chi-square, Student's t-test and non-parametric tests.

As shown in Table 1, the hospitalization rate of patients infected with the SARS-CoV-2 Omicron variant in different vaccine dose groups was different (76.3% vs. 70.1% vs. 67.9% vs. 62.0%, *p* < 0.001). The admission rate among patients receiving three vaccine doses was significantly less than the other dose groups (62.0 vs. 70.1%, *p* < 0.001; 62.0 vs. 67.9%, *p* = 0.024). We investigated the vaccine timing from vaccination to infection with the SARS-CoV-2 Omicron variant, the first dose at 253 days (range, 199–295 days), the second dose at 100 days (range,79–120 days), and the third dose at 27 days (range, 22–35 days). We further compared the length of hospitalization among patients who had one, two, and three vaccine doses. The length of hospitalization was significantly shorter in vaccinated patients than that in unvaccinated patients (16.60 ± 7.83 vs. 12.41 ± 6.64, *p* = 0.001). Viral persistence in patients who received one, two, and three vaccine doses were shortened than that in unvaccinated patients (10.49 ± 4.40 vs. 11.04 ± 5.76 vs. 10.34 ± 5.45 vs. 12.41 ± 6.64, *p* = 0.010). Viral persistence in patients who received three vaccine doses was significantly shorter than in unvaccinated patients (12.41 ± 6.64 vs. 10.63 ± 5.55, *p* = 0.032; Table 1).

As the number of vaccine doses increased, the incidence of clinical symptoms, such as fever, fatigue, and muscle soreness, gradually decreased; however, the incidence of cough and rhinobyon symptoms increased. The incidence of asymptomatic patients increased and the ordinary type decreased as the number of vaccine doses increased (Table 1).

**Figure 1:**
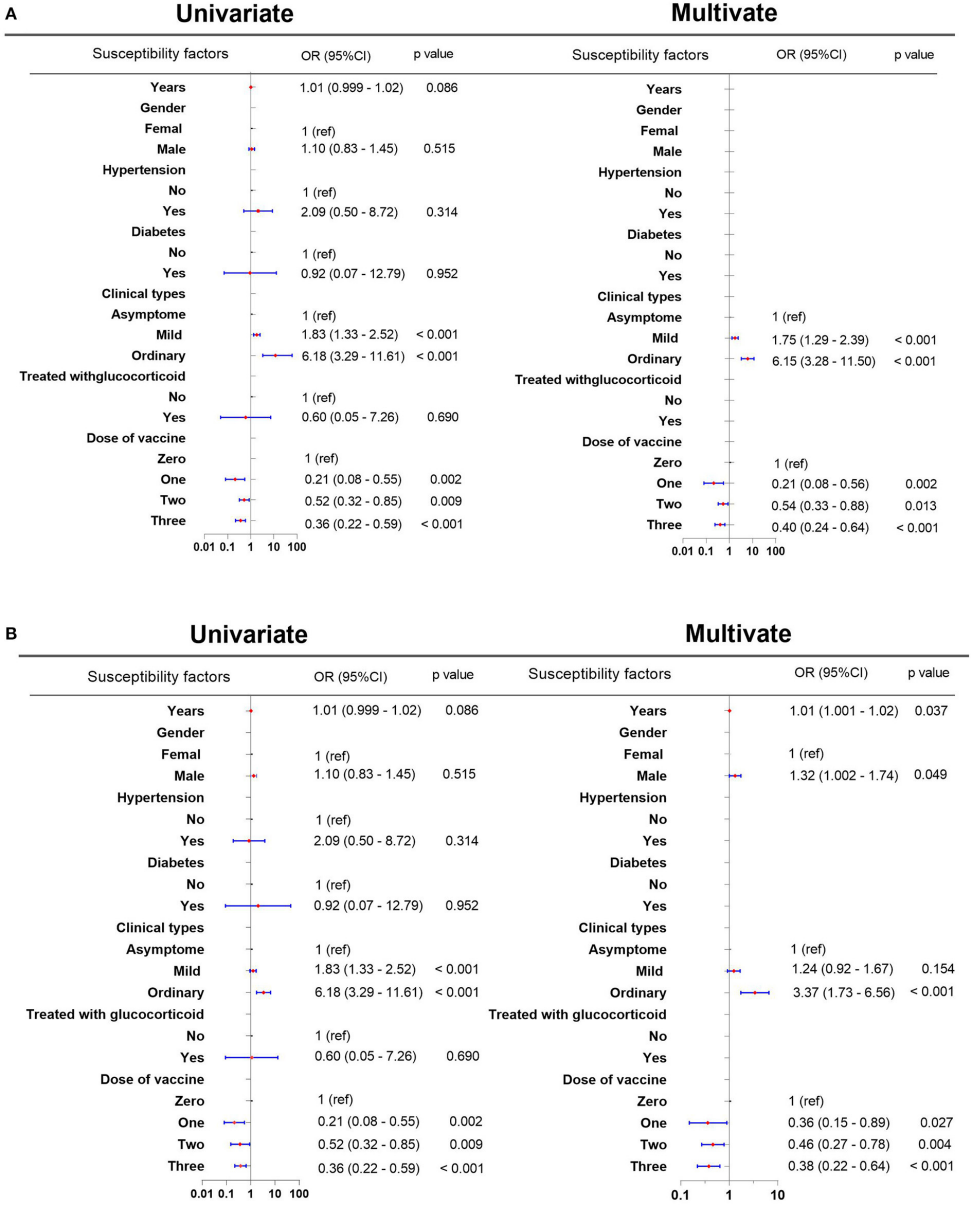
Susceptibility factors associated with prolonged hospitalization and persistent virus in the sputum. **(A)** Susceptibility factors associated with prolonged hospitalization. **(B)** Susceptibility factors associated with the prolonged persistence of the virus in the sputum. CI, confidence interval; OR: odds ratio.

Because the mean length of hospitalization was 14 days (range, 1–59 days), we defined prolonged hospitalization as the length of hospitalization of >14 days. We defined the persistence of the virus in the sputum as the duration of detectable Omicron SARS-CoV-2 RNA variant in sputum of >11 days because the mean time was 11 days (range, 1–33 days). To identify the susceptibility factors associated with prolonged hospitalization and viral persistence in the sputum, the variables entered into the multivariate analysis were sex, years of patients with COVID-19, co-morbid hypertension, co-morbid diabetes, glucocorticoid use, clinical types of COVID-19, and the number of vaccine doses. Based on univariate and multivariate analyses, the number of vaccine doses and the clinical types of COVID-19 were associated with prolonged hospital length of stay and viral persistence in the sputum (Figure 1). Based on the multivariate analysis, one vaccine dose [odds ratio (OR): 0.21, 95% confidence interval (CI): 0.08–0.56, *p* = 0.002], two vaccine doses (OR: 0.54, 95% CI: 0.33–0.88, *p* = 0.013), and three vaccine doses (OR: 0.40, 95% CI: 0.24–0.64, *p* < 0.001) shortened the length of hospital stays in patients with three vaccine doses than that in unvaccinated patients (Figure 1A). The persistence of the virus in the sputum was significantly shortened in patients who received one vaccine dose (OR: 0.36, 95% CI: 0.15–0.89, *p* = 0.027), two vaccine doses (OR: 0.46, 95% CI: 0.27–0.78, *p* = 0.004), and three vaccine doses (OR: 0.38, 95% CI: 0.22–0.64, *p* < 0.001) than that in unvaccinated patients (Figure 1B).

## Discussion

The emergence of the new Omicron variant has raised serious public health concerns due to multiple mutations, immune escape, and an unprecedented rate of rapid spread (10). Some reports have shown that the Omicron variant results in less severe symptoms and a lower mortality rate than other SARS-CoV-2 variants (6, 11); however, the mortality rate of patients infected with the Omicron variant is not insignificant according to Hong Kong data (3). The mortality rate among patients who received three vaccine doses was decreased when compared to patients who were not vaccinated (0.03 vs. 3.2%); however, the clinical impact of COVID-19 vaccination in non-severe and non-critical patients infected with the Omicron virus is not known. In the current study, we analyzed the clinical data from patients infected with the Omicron variant in a retrospective cohort of 5,713 hospitalized patients in the Zhejiang Province of China.

The length of hospitalization and viral persistence among patients who were vaccinated were shortened than that in unvaccinated patients. Although the length of hospitalization and viral persistence in patients who received three vaccine doses were not different from the corresponding outcomes in those who received only one or two doses, the multivariate regression analysis supported the conclusion that vaccination with a third dose was a significant factor in shortening hospital stay and viral clearance time. Some reports have shown that vaccine effectiveness against SARS-CoV-2 wanes over time (12, 13). We showed that vaccination was a more effective way to decrease some symptoms, clinical ordinary types, and admission rate, and shorten hospitalization and viral persistence in the sputum, compared with those without vaccination, which is consistent with previous reports (14, 15). Although our study subjects were limited to patients who sought evaluation and treatment at a medical institute, all infected individuals in the community will undergo SARS-CoV-2 RNA testing in China when diagnosed with COVID-19 and all confirmed patients with COVID-19 will be quarantined in isolation wards or Fangcang hospitals. This study was performed in China and those receiving multiple inactivated, identical vaccines, and not compared with mRNA- or viral vector-based vaccines against SARS-CoV-2 with respect to severity and effect. In the current study, we did not analyze the side effects of the vaccine. Because, some patients who had side effects after vaccination but were not infected with the SARS-CoV-2 Omicron variant may be excluded, which may lead to an underestimation of the vaccine's side effects.

In conclusion, vaccination was an effective way to protect people against the Omicron variant infection. Indeed, on the premise of the current routine recommendation of vaccination, three vaccines were necessary for people to be protected against the Omicron variant. Furthermore, the efficacy of more than three vaccine doses specifically targeted against Omicron and its most prevalent subvariants warrant further study.

## Data availability statement

The datasets presented in this study can be found in online repositories. The names of the repository/repositories and accession number(s) can be found in the article/supplementary material.

## Ethics statement

The studies involving human participants were reviewed and approved by the Ethics Committee of the First Affiliated Hospital of Zhejiang University School of Medicine. The patients/participants provided their written informed consent to participate in this study.

## Author contributions

HZ, YS, and JS conceived and designed the study. HZ, WY, and XY collected data and analyzed the data. HZ and WY draw the manuscript. YS and JS revised the manuscript. All authors approved the final version of the manuscript, including the authorship list.
